# A Novel Variant of GP9 Gene Resulting in Bernard-Soulier Syndrome: A Case Report

**DOI:** 10.7759/cureus.76363

**Published:** 2024-12-25

**Authors:** Badriah G Alasmari, Sameer M Alqahtani, Ali Alabbas, Muhammad Saeed, Lina Elzubair, Fahad S Alqahtani, Hind R Alasmari, Wafa A Alrezqi, Saeed M Al-Tala

**Affiliations:** 1 Pediatrics, Armed Forces Hospital Southern Region, Khamis Mushait, SAU; 2 Pediatrics, Najran General Hospital, Najran, SAU; 3 Pediatric Neurology, Armed Forces Hospital Southern Region, Khamis Mushait, SAU; 4 Pathology, Armed Forces Hospital Southern Region, Khamis Mushait, SAU; 5 Pediatric Medicine, Armed Forces Hospital Southern Region, Khamis Mushait, SAU; 6 Faculty of Medicine, Albaha University, Albaha, SAU; 7 Pediatric, Armed Forces Hospital Southern Region, Khamis Mushait, SAU

**Keywords:** bernard-soulier syndrome (b.s.s), immune thrombocytopenia (itp), (nsaids) nonsteroidal anti-inflammatory drugs, pbs( peripheral blood smear ), whole exome sequencing (wes)

## Abstract

Bernard-Soulier syndrome (BSS) is a rare autosomal recessive condition that is defined by low platelet count and platelet dysfunction characterized by the absence or dysfunction of the *GpIb V/IX* complex on the platelet surface. It is characterized by large defective platelets and thrombocytopenia. BSS is usually presented early in life. Clinical manifestations of BSS include bleeding that affects both the skin and mucous membranes, including purpura, nasal, and gum bleeding. Also, it can present with symptoms, such as menometrorrhagia or gastrointestinal bleeding. Herein, we describe the case of a five-year-old girl with a novel variant of the *GP9 *gene resulting in BSS type C, with silent clinical manifestation with the exception of a pattern of easy bruising.

## Introduction

Bernard-Soulier syndrome (BSS) was initially reported by Bernard and Soulier in 1948 as an inherited bleeding disorder characterized by low platelet count and large platelets [1]. BSS is a unique platelet disorder inherited through autosomal recessive transmission and identified by low levels, lack, or malfunction of the platelet *GpIb/V/IX* complex. BSS is defined by mild to moderate thrombocytopenia and is marked by reduced platelet levels, adherence, atypical use of prothrombin, and diminished survival of oversized platelets [2]. Clinical manifestations of BSS often include mucocutaneous bleeding (e.g., purpura), gastrointestinal bleeding, menorrhagia, and bleeding through the nose and gums. This presentation is quite similar to those of other conditions and platelet disorders [3]. BSS is caused by mutations in *GP1BA (GPIbα), GP1BB (GPIbß), and GP9 (GPIX)*. Four genes are responsible for encoding the subunits of the *GPIb-IX-V *complex [4].

Herein, we describe the case of a five-year-old girl with chronic immune thrombocytopenic purpura (ITP). Peripheral blood smear (PBS) showed giant platelets, and platelet function testing supported a diagnosis of BSS. Whole exome sequence (WES) genetic study revealed a *GP9 *gene mutation, confirming the diagnosis of BSS type c.

## Case presentation

A five-year-old girl delivered via spontaneous vaginal delivery with no neonatal intensive care unit (NICU) admission presented to our department. The patient had normal growth and development. When the patient was four years old, the mother took the patient for routine lab work, including a complete blood count, which accidentally found a platelet count of 75 × 10^9^/L. Repeat sampling also showed a platelet count of 75 × 10^9^/L. She was brought to the hospital with a previous history of easy bruising after falling down while playing, but no previous history of epistaxis or mucocutaneous bleeding and no gastrointestinal bleeding.

Initial investigations in the hospital showed a platelet count range of 40-50 × 10^9^/L. While monitoring the patient, her platelet count first dropped to 15 × 10^9^/L and then to 7 × 10^9^/L, with no obvious cause. The patient was not taking any nonsteroidal anti-inflammatory drugs, and she had no family history of platelet dysfunction or bleeding tendencies. Therefore, the patient was diagnosed with chronic ITP and received intravenous immunoglobulin for seven days with hydrocortisone intravenously (IV). She was then discharged on oral prednisolone with no major bleeding at the time. Her last platelet count was 65 × 10^9^/L with follow-up in the clinic.

During our patient’s illness and before her final diagnosis, her mother was pregnant and delivered a baby boy who was admitted to the NICU due to thrombocytopenia. The patient’s brother’s platelet count was 20 × 10^9^/L. The parents of both children were first-degree consanguineous.

During clinical examination of the patient, she was vitally stable, conscious, alert, and maintaining normal oxygen saturation in ambient air with no respiratory distress, not in pain, had no dysmorphic features, jaundice, or cyanosis, and her growth parameters were within normal ranges. The patient had no petechiae, purpura, bruising, ecchymosis, or observable active bleeding, and no joint swelling or bone tenderness. Neurological examination was normal. A gastrointestinal examination revealed a soft, lax abdomen and no organomegaly. Further systemic examination was unremarkable.

Laboratory investigation revealed a platelet count of 21 × 10^9^/L (normal range: 150-450 × 10^9^/L). Her liver function test, renal profile, and inflammatory markers were normal.

**Figure 1:**
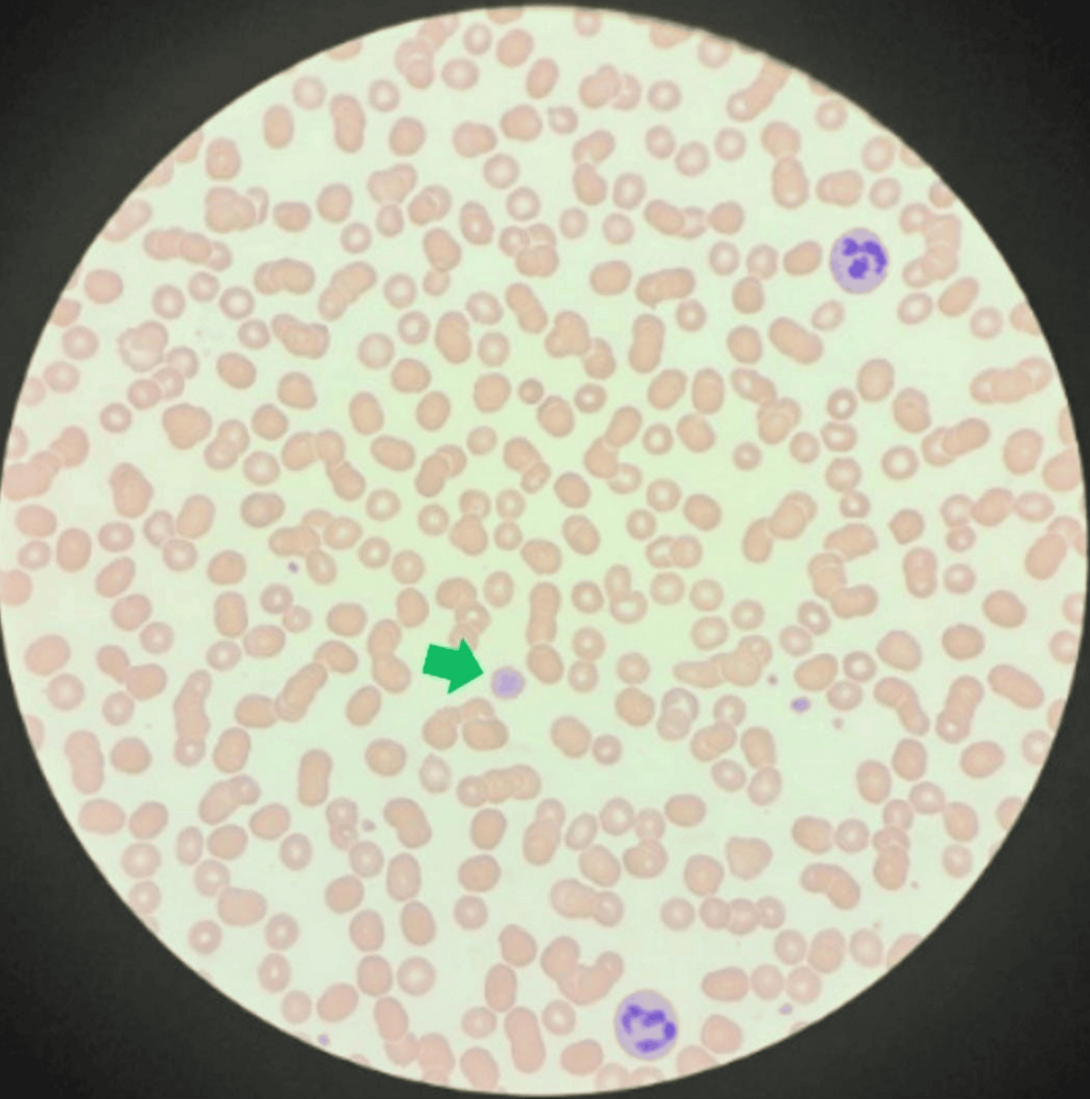
Peripheral blood smear x 100 with Wright-Giemsa staining showing moderate thrombocytopenia with occasional large and giant platelets (green arrow), and a manual platelet count of 70 x109/L.

**Figure 2:**
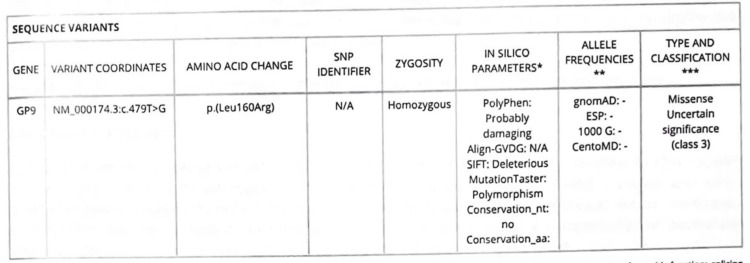
Whole exome sequence result showing GP9 gene mutation.

PBS revealed giant platelets, as shown in Figure 1. Platelet function testing supported a diagnosis of BSS, with specific platelet membrane glycoprotein demonstrating elevated expression of *CD61 (GpIIIa)* and *CD41 (GpIIB)*. Conversely, there was an absence of expression for *CD42a (GPIX)* and *CD42B (GpIb)*. These findings are consistent with BSS. WES showed a *GP9* gene mutation, confirming a diagnosis of BSS type c, as shown in Figure 2. Both parents were carriers of the same *GP9* gene mutation. The patient received follow-up in our pediatric hematology clinic; her last platelet count was 160 × 109/L, she was clinically stable, and her condition improved. Similarly, the patient’s brother was found to have the same *GP9* gene mutation, resulting in a diagnosis of BSS type c.

## Discussion

We report the case of a five-year-old girl with a mild phenotype of GP9 gene mutation resulting in a diagnosis of BSS type c. BSS has an occurrence of approximately one in every one million live births; however, the disease is under-reported because it is frequently misdiagnosed [4]. In one study conducted in Iran involving 97 BSS patients, 81% of cases reported consanguineous marriage [5].

Currently, there is no curative therapy for BSS, with only supportive management being available, such as platelet transfusion when required. BSS patients often make lifestyle changes to manage symptoms, such as avoiding contact sports, and they must notify their doctor prior to any surgical procedure. If BSS patients receive proper education, as well as supportive medical care and management, life expectancy is generally not affected [6-8].

The family of the patient in this case report was educated about BSS and given clinical instruction for when they must seek treatment at the emergency room (e.g., trauma, mucocutaneous bleeding, or gastrointestinal bleeding). For the management of BSS cases involving bleeding and thrombosis, it is crucial to focus on prevention. First, it is important to educate both the patient and their family about bleeding risks and prevention, such as engaging in safe activities, avoiding high-risk sports, and applying pressure to wounds. In addition, patients must be aware of medications that can cause bleeding or interfere with clotting, such as heparin, warfarin, aspirin, ibuprofen, and naproxen. Families with a history of consanguinity or carriers of certain genetic conditions are at higher risk for their babies to develop BSS; therefore, it is essential to provide them with counseling before and during pregnancy. Mothers with BSS should be closely monitored for any signs of bleeding during pregnancy and after childbirth. If recombinant factor VII (rFVIIa) is used as a medication, additional measures should be considered to prevent blood clots [9]. During childbirth, it is best to avoid using neuraxial anesthesia due to the risk of unstable blood pressure in the patient. If necessary, safer options include tranexamic acid, uterotonics, rFVIIa, and human leukocyte antigen matching [10].

## Conclusions

BSS presents with low platelet count and dysfunction of the GpIb V/IX complex. It is inherited in an autosomal recessive manner, with clinical manifestation characterized by mucocutaneous or gastrointestinal bleeding. Diagnosis of BSS is essential for appropriate treatment and must be made through clinical and laboratory investigations, such as PBS, platelet function tests, and genetic studies.

BSS is managed supportively with platelet transfusion and IV immunoglobulin when needed. In cases of active bleeding, tranexamic acid should be given with rFVIIa. The role of geneticists and preventive medicine is essential for parent education and family segregation for future offspring. Healthy lifestyles should be promoted through education via media and scientific symposia and should be open to the general public.
